# Genome Sequence of *Rhodococcus* sp. 4J2A2, a Desiccation-Tolerant Bacterium Involved in Biodegradation of Aromatic Hydrocarbons

**DOI:** 10.1128/genomeA.00592-15

**Published:** 2015-06-04

**Authors:** Maximino Manzanera, Cristina García-Fontana, Juan Ignacio Vílchez, Jesús González-López

**Affiliations:** Institute for Water Research and Department of Microbiology, University of Granada, Granada, Spain

## Abstract

The genome sequence for *Rhodococcus* sp. 4J2A2, a newly described desiccation-tolerant strain that removes aromatic hydrocarbons, is reported here. The genome is estimated to be around 7.5 Mb in size, with an average G+C content of 60.77% and a predicted number of protein-coding sequences of 6,354.

## GENOME ANNOUNCEMENT

*Rhodococcus* sp. 4J2A2 is a desiccation-tolerant Gram-positive bacterium belonging to the *Actinobacteria* phylum and the *Nocardiaceae* family, and it was isolated from the *Nerium oleander* rhizosphere (1). The genome sequences of other desiccation-tolerant microorganisms have been reported (2, 3), including that of the recently described new species *Arthrobacter siccitolerans* 4J27 (4). These microorganisms produce different compounds, substances known as xeroprotectants (5), in response to changes in osmotic conditions and water activity (1). These compounds, produced to protect essential biomolecules and cell integrity, allow the cell to tolerate extremely low concentrations of water and other chemical insults (6, 7).

Species of the genus *Rhodococcus* have been described as efficient removers of pollutants, particularly aromatic organic compounds (8). Here, we report the whole-genome sequence of *Rhodococcus* sp. 4J2A2, obtained with pyrosequencing technology implemented in the 454 Life Science-Roche platform with a combined approach based on shotgun and 8-kb mate pair sequencing (Lifesequencing SL, Valencia, Spain) (9). This technology was used to obtain a total of 222,955 reads. The average read length for the shotgun sequencing approach was 661 nucleotides, rendering 123,125 sequences. The average read length for the mate pair sequencing strategy was 419.72 bases. The total number of sequenced bases was 123,294,461, representing a sequencing depth of around 16×. For *de novo* assembly, Newbler Assembler version 2.6 was used with default parameters. This assembly yielded 60 contigs, 45 of which were >500 bp. The *N*_50_ of the contig assembly was 375,535 bp, and the largest contig was 890,470 bp. Mate pair information indicated that most of these contigs were ordered in four scaffolds, the largest comprising 4,858,281 bp. The estimated genome size of 7.5 Mb was deduced from this combination of scaffolds and contigs. Gap-spanning clones and PCR products were used to attempt gap closure, and putative coding sequences were predicted. Genes were annotated with a pipeline implemented at Lifesequencing, and protein-coding sequences (CDS) were predicted with Glimmer (10–12), RNAmmer (13), tRNAscan (14, 15), and BLAST (16, 17) in combination. Most of the contigs used to obtain complete genomic information for *Rhodococcus* 4J2A2 are contained on four scaffolds, with an average G+C content of 60.77%. The genome was found to contain 6,354 protein-coding genes, 4 rRNA operons, and 51 tRNA genes.

On the basis of this genome sequence, we propose the presence of pathways for the catabolism of chloroalkanes and chloroalkenes, such as *cis*- and *trans*-dichloropropene, trichloroethane, and tetrachloroethene via pyruvate, glyoxylate, dicarboxylate, and methane metabolism. We also propose the presence of pathways for the metabolism of aromatic hydrocarbons, such as toluene, xylene, benzoate, and phthalate, and polycyclic hydrocarbons, such as fluorene, anthracene, phenanthrene, pyrene, benzo[*a*]pyrene, and naphthalene, and some of its derivate compounds.

The complete genome sequence of *Rhodococcus* sp. 4J2A2 will contribute to the development of biotechnological applications in the field of bioremediation, particularly for the removal of pollutants in arid regions (6, 18).

### Nucleotide sequence accession numbers.

The complete genome sequence of *Rhodococcus* sp. 4J2A2 has been deposited in the TBL/EMBL/GenBank databases under the accession numbers CEDU01000001 to CEDU01000060.
